# Total alkaloids from *Coptis chinensis* Franch ameliorate hyperlipidemia and hepatic steatosis via dual pathway modulation of AMPK/SREBP-1c and PPARα/LXRα in mice

**DOI:** 10.3389/fphar.2025.1662325

**Published:** 2025-10-31

**Authors:** Xiangyang Li, Xiaomin Zhang, Nina Wei, Haibo Peng, Xianli Niu

**Affiliations:** ^1^ Guangdong Provincial People’s Hospital, Zhuhai Hospital (Jinwan Central Hospital of Zhuhai), Zhuhai, Guangdong, China; ^2^ Guangdong Jiangmen Chinese Medicine College, Jiangmen, Guangdong, China; ^3^ Zhuhai Campus of Zunyi Medical University, Zhuhai, Guangdong, China

**Keywords:** Coptis chinensis, alkaloids, hyperlipidemia, hepatic steatosis, murine model, AMPK signaling

## Abstract

**Introduction:**

Hyperlipidemia and its associated hepatic steatosis pose significant global health burdens, necessitating novel therapeutic strategies. *Coptis chinensis* Franch total alkaloids (TAC) exhibit lipid-modulating properties, but their mechanistic underpinnings remain incompletely elucidated. We aim to evaluate the therapeutic efficacy and molecular mechanisms of TAC in a murine model of hyperlipidemia-associated NAFLD.

**Materials and methods:**

High-fat diet (HFD)-fed C57BL/6 mice received TAC (2.5, 5.0, 10.0 g/L) or simvastatin for 2 weeks. Metabolic parameters, serum lipid profiles, hepatic function markers, and histopathology were systematically analyzed. Molecular pathways were interrogated through qPCR, Western blot, and pharmacological inhibition of AMPK (Compound C) and PPARα (GW6471).

**Results:**

TAC treatment demonstrated significant dose-dependent improvements across multiple parameters. Compared to HFD controls, TAC reduced body weight by 21.3% and liver index by 18.7%, while lowering fasting blood glucose levels by 32.4%. Serum analyses showed substantial reductions in total cholesterol (46.2%), triglycerides (38.5%), and LDL-cholesterol (52.1%), accompanied by a 29.8% increase in HDL-cholesterol. Hepatic function improved markedly, with ALT and AST levels decreasing by 57.3% and 49.6% respectively. Histopathological examination revealed a 68.4% reduction in hepatic lipid accumulation. At the molecular level, TAC treatment resulted in a 2.7-fold increase in AMPK phosphorylation while significantly reducing HMGCR expression by 63.1% and nuclear SREBP-1c levels by 71.5%. Concurrently, TAC upregulated PPARα and LXRα expression by 3.1-fold and 2.4-fold respectively, leading to enhanced expression of lipolytic enzymes LPL and HL by 2.8-fold and 2.1-fold. These beneficial effects were completely abolished by co-treatment with pathway-specific inhibitors.

**Conclusion:**

TAC ameliorates hyperlipidemia and hepatic steatosis through dual modulation of AMPK/SREBP-1c-mediated lipid synthesis and PPARα/LXRα-driven lipolysis, presenting a multifaceted therapeutic approach for metabolic disorders.

## 1 Introduction

With the global advancement of living standards, hyperlipidemia and its associated disorders such as non-alcoholic fatty liver disease (NAFLD) have emerged as paramount health challenges of international concern (Jiang et al., 2025). Hyperlipidemia, defined as the abnormal elevation of blood lipid levels, typically involves increases in cholesterol and triglycerides (Harada-Shiba et al., 2023). This metabolic derangement is intricately linked to a spectrum of health issues, most notably NAFLD and cardiovascular-cerebrovascular diseases (Nehaoua et al., 2025). NAFLD, a prevalent chronic liver disorder, is characterized by excessive hepatic fat accumulation in the absence of significant alcohol consumption (Guo et al., 2022). Emerging evidence indicates that hyperlipidemia serves as a critical risk factor for NAFLD, instigating hepatic lipid metabolic dysregulation that promotes excessive fat deposition in the liver (Nehaoua et al., 2025). Concurrently, hyperlipidemia maintains a profound association with the pathogenesis of cardiovascular and cerebrovascular diseases (Pedro-Botet et al., 2024; Xu et al., 2024). Elevated low-density lipoprotein cholesterol (LDL-C) is recognized as a pivotal contributor to atherosclerosis, the primary pathological substrate of cardiovascular diseases (Pedro-Botet et al., 2024). The excessive accumulation of LDL-C induces lipid deposition within arterial walls, culminating in the formation of atherosclerotic plaques that may precipitate severe cardiovascular events such as myocardial infarction or stroke (Xu et al., 2024).

In the realm of hyperlipidemia therapy, beyond conventional pharmacotherapies such as statins, innovative therapeutic modalities have been developed in recent years (Han et al., 2019). *Coptis chinensis* Franch (TAC), a revered traditional Chinese herbal medicine, has garnered significant attention for its bioactive constituents, total alkaloids from TAC, in the management of hyperlipidemia and fatty liver (Coppinger et al., 2024). Preclinical and clinical investigations have demonstrated that berberine (BBR), a key alkaloid component of TAC, exhibits remarkable efficacy in regulating blood lipid profiles and ameliorating NAFLD (Yang et al., 2024). Mechanistically, BBR modulates lipid and glucose metabolic dysregulations in NAFLD through direct targeting of the AKR1B10 protein, thereby improving hepatic steatosis, insulin resistance, and triglyceride homeostasis (Yang et al., 2024). Additionally, BBR exerts cardioprotective effects through multifaceted mechanisms, leveraging its antioxidant, anti-inflammatory, and lipid-lowering properties to mitigate the risk of cardiovascular events (An et al., 2022). BBR alleviates atherosclerotic progression by modulating the gut microbiota to suppress the production of trimethylamine (TMA) and trimethylamine-N-oxide (TMAO) (Li et al., 2021). Emerging research further indicates that BBR ameliorates glucose-lipid metabolic disorders and suppresses vascular inflammation by enhancing the interaction between KLF16 and PPARα, thereby attenuating diabetic atherosclerosis (Man et al., 2022). In the context of NAFLD therapy, BBR significantly reduces hepatic lipid accumulation and inflammatory responses by regulating multiple signaling pathways, including those governing fatty acid synthesis and metabolism, bile acid homeostasis, as well as inflammation- and fibrosis-related gene expression (Wang et al., 2021). Collectively, these findings underscore the multi-targeted pharmacological properties of berberine in managing metabolic disorders and cardiovascular diseases, heralding promising clinical applications. Notwithstanding these advancements, the efficacy of TAC in hyperlipidemia-combined NAFLD and its underlying molecular mechanisms remain largely unelucidated.

Thus, the present study aims to establish a murine model of NAFLD complicated by hyperlipidemia and fatty liver, investigate the effects of TAC on glycemic-lipid parameters, hepatic function, and pathological alterations in mice, and explore the therapeutic value and potential molecular mechanisms of TAC in managing hyperlipidemia with fatty liver. These efforts seek to provide scientific rationale for the clinical application of *C. chinensis* Franch in related disease entities.

## 2 Materials and methods

### 2.1 Experimental materials

SPF-grade healthy 5-week-old male C57BL/6 mice (body weight ∼30 g) were procured from Zhuhai Baishitong Biotechnology Co., Ltd. (License No. SCXK [Yue] 2020-0051). The total alkaloids from Coptis chinensisFranch (TAC) were obtained from Guangzhou Kangcheng Biotechnology Co., Ltd. (Guangzhou, China). The product is a standardized botanical extract with a certified total alkaloid content of ≥98% as determined by high-performance liquid chromatography (HPLC). The quantitative composition of the major alkaloidal constituents is as follows: Berberine (∼75%), Coptisine (∼10%), Palmatine (∼6%), and Epiberberine (∼3%). Experimental reagents included commercial assay kits for total cholesterol (TC), triglyceride (TG), low-density lipoprotein cholesterol (LDL-C), high-density lipoprotein cholesterol (HDL-C), alanine aminotransferase (ALT), aspartate aminotransferase (AST), hepatic lipase (HL), lipoprotein lipase (LPL), and HMG-CoA reductase (HMGCR) (Nanjing Jiancheng Bioengineering Institute), as well as simvastatin (Hangzhou Merck Sharp & Dohme Pharmaceuticals Co., Ltd.). Primary antibodies against HMGCR, AMP-activated protein kinase (AMPK), phosphorylated AMPK (p-AMPK, Thr172), sterol regulatory element-binding protein-1c (SREBP-1c), LPL, HL, peroxisome proliferator-activated receptor α (PPARα), peroxisome proliferator-activated receptor γ (PPARγ), liver X receptor α (LXRα), and glyceraldehyde-3-phosphate dehydrogenase (GAPDH) were obtained from Abcam. Instruments included an XS205DU analytical balance (Ohaus, United States), an automatic tissue dehydrator (Leica, Germany), and a multi-mode microplate reader (BioTek, United States).

### 2.2 Establishment of hyperlipidemia-fatty liver model

SPF-grade healthy C57BL/6 male mice were randomly assigned to six groups (*n* = 9 per group): normal control, model control, TAC high-, medium-, and low-dose groups, and simvastatin positive control group. After a 1-week adaptive feeding period, the normal group was fed a basal diet, while the other five groups received a high-fat diet (HFD). The HFD regimen was composed of 60% kcal% fat (primarily lard), 20% kcal% carbohydrate, and 20% kcal% protein, supplemented with 1.25% cholesterol and 0.5% sodium cholate to accelerate the development of dyslipidemia and robust hepatic steatosis. This diet was chosen because its composition—high in saturated fats and cholesterol—mimics the excessive intake of energy-dense, processed foods that is a hallmark of the Western diet in humans. The mice were maintained on this diet for a total of 6 weeks (4 weeks for model establishment +2 weeks for drug intervention) to ensure the full development of the metabolic phenotype. Body weight and blood glucose were monitored every 4 days. Following 4 weeks of feeding, mice were fasted for 8 h, and tail blood was collected to measure serum TC, TG, LDL-C, HDL-C, AST, and ALT. The hyperlipidemia-fatty liver model was validated when these indices showed significant differences from the normal group (*P < 0.05). Mice in the normal and model control groups were gavaged with 0.9% NaCl, the simvastatin group with 0.3 mg/mL simvastatin (10 mL/kg body weight), and the TAC groups with 2.5 g/L, 5.0 g/L, or 10.0 g/L TAC for 2 consecutive weeks. Physiological parameters (body weight, blood glucose, lipid profiles) were recorded periodically.

### 2.3 Measurement of physiological indices and lipid profiles

Post-treatment, body weight was measured, and blood glucose was determined via tail prick. Mice were euthanized by cervical dislocation, and livers were excised, rinsed with 0.9% NaCl, blotted dry, and weighed to calculate the liver coefficient using the formula: Liver coefficient (%) = (liver wet weight/body weight) × 100%.

### 2.4 Biochemical analysis of lipids and hepatic function

Serum was separated by centrifugation post-treatment, and TC, TG, LDL-C, HDL-C, ALT, AST, LPL, HL, and HMGCR levels were assayed per kit protocols. Atherosclerosis indices (AI1, AI2) and coronary heart risk index (R-CHR) were calculated as: AI1 = (TC - HDL-C)/HDL-C, AI2 = LDL-C/HDL-C, R-CHR = TC/HDL-C.

### 2.5 Histopathological evaluation of liver tissue

Following euthanasia, liver lobules were fixed in 4% paraformaldehyde for 12 h, dehydrated, embedded in paraffin, and sectioned into 5-μm slices. Sections were stained with hematoxylin-eosin (H&E) according to standard protocols and observed under an optical microscope for pathological alterations. For quantitative assessment of hepatic steatosis, digital image analysis was performed. Five randomly selected fields per liver section (*n* = 5 mice per group) were captured at ×200 magnification. The area percentage of lipid droplets relative to the total hepatocyte area was quantified in a blinded manner using ImageJ software (United States). The percentage of the thresholded area (lipid droplets) relative to the total area of the parenchyma was then calculated.

### 2.6 RT-qPCR analysis

Total RNA was extracted from 0.1 g of liver tissue using TRIzol^®^ Reagent (Invitrogen). After chloroform phase separation, RNA was precipitated with isopropanol, washed twice with 75% ethanol (DEPC-treated), and dissolved in 20 μL nuclease-free water. Reverse transcription was performed using PrimeScript™ RT Master Mix (Takara), and cDNA was diluted to 25 ng/μL. mRNA levels were quantified by SYBR^®^ Green Master Mix (Applied Biosystems) via ΔΔCt analysis. RT-qPCR primers were as follows: -HMGCR: F: 5′-GGC​ATT​TGA​CAG​CAC​TAG​CA-3′, R: 5′-CTT​TGC​ATG​CTC​CTT​GAA​CA-3′; -SREBP-1c: F: 5′-GAT​GTG​CGA​ACT​GGA​CA-3′, R: 5′-CAT​AGG​GGG​CGT​CAA​ACA​G-3′; -SREBP-2: F: 5′-AAG​TCT​GGC​GTT​CTG​AGG​AA-3′, R: 5′-CAC​AAA​GAC​GCT​CAG​GAC​AA-3′; -LDLR: F: 5′-CTC​GCT​GGT​GAC​TGA​AAA​CA-3′, R: 5′-CAA​AGG​AAG​ACG​AGG​AGC​AC-3′; -FAS: F: 5′-GGA​GGT​GGT​GAT​AGC​CGG​TAT-3′, R: 5′-TGG​GTA​ATC​CAT​AGA​GCC​CAG-3′; -ACC: F: 5′-AGG​TGG​TAC​GGA​TGG​CTG​CTC-3′, R: 5′-GAC​GGT​GCT​GGA​CGC​TGT​TG-3′; -LPL: F: 5′-TTG​CCC​TAA​GGA​CCC​CTG​AA-3′, R: 5′-ACA​GAG​TCT​GCT​AAT​CCA​GGA​AT-3′; -HL: F: 5′-GAC​GGG​AAG​AAC​AAG​ATT​GG-3′, R: 5′-GGC​ATC​ATC​AGG​AGA​AAG​G-3′.

### 2.7 Western blot analysis

Liver tissue (0.1 g) was rinsed with pre-cooled PBS, minced, and lysed in RIPA buffer containing 1 mM PMSF and phosphatase inhibitors on ice for 30 min. Lysates were centrifuged at 12,000×g for 15 min at 4 °C, and supernatants were collected. Protein concentration was determined by Pierce™ BCA Kit. Proteins were separated by SDS-PAGE and transferred to PVDF membranes, which were blocked with 5% skim milk for 2 h at RT, then incubated with primary antibodies at 4 °C overnight. After TBST washing (3 × 10 min), membranes were probed with HRP-conjugated secondary antibodies for 1 h at RT. Bands were detected via ECL chemiluminescence (Thermo Fisher), and densitometry was analyzed by ImageLab software (v6.1, Bio-Rad) using GAPDH as a loading control.

### 2.8 Statistical analysis

Data are presented as mean ± standard deviation (SD) and analyzed by one-way analysis of variance (ANOVA) using SPSS 17.0. Statistical significance was set at *P < 0.05.

## 3 Results

### 3.1 Effects of TAC on the body weight, liver wet weight, liver coefficient, and blood glucose of mice

**FIGURE 1:**
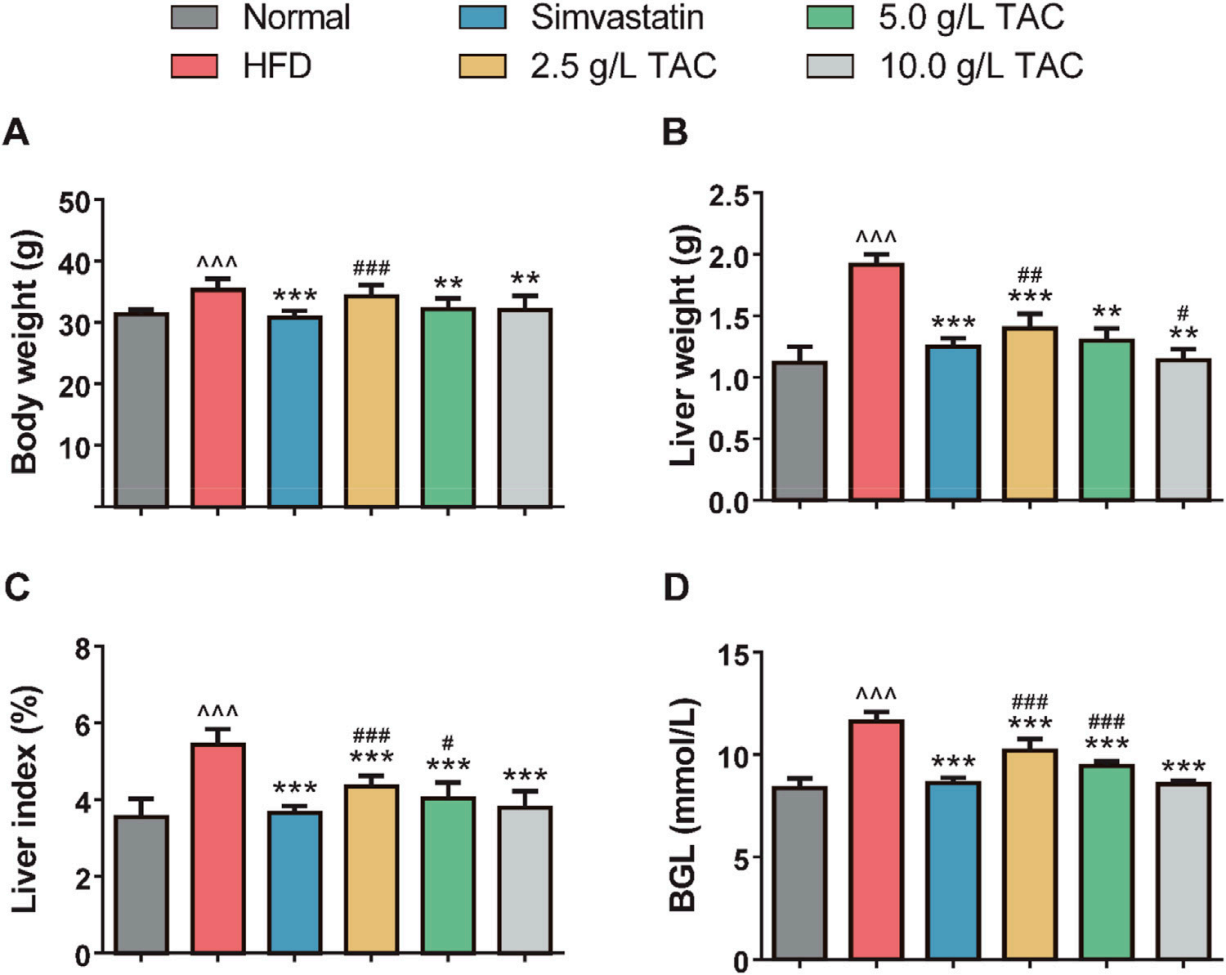
Effects of TAC on physiological indices in hyperlipidemic fatty liver mice. Effects of different doses of TAC on **(A)** body weight change, **(B)** liver wet weight, **(C)** liver index (liver wet weight/body weight × 100%), and **(D)** fasting blood glucose level (BGL). Data are presented as mean ± standard deviation (SD), *n* = 9. ^^^P < 0.001 vs. Normal group; **P < 0.01, ***P < 0.001 vs. HFD group; ^#^P < 0.05, ^##^P < 0.01, ^###^P < 0.001 vs. simvastatin group.

As shown in Figure 1, compared with the normal group, the model control group exhibited significant increases in body weight, liver wet weight, liver coefficient, and blood glucose, indicating successful establishment of the hyperlipidemia-combined fatty liver model in mice. In contrast, the simvastatin group and TAC treatment groups (all doses) showed significant reductions in these parameters (*P < 0.05), with a dose-dependent response to TAC, suggesting therapeutic effects of TAC on the liver of mice with hyperlipidemia and fatty liver. Notably, the liver wet weight in the high-dose TAC group was significantly lower than that in the simvastatin group (*P < 0.05).

### 3.2 Effects of TAC on blood lipid levels in mice with hyperlipidemia and fatty liver

**FIGURE 2:**
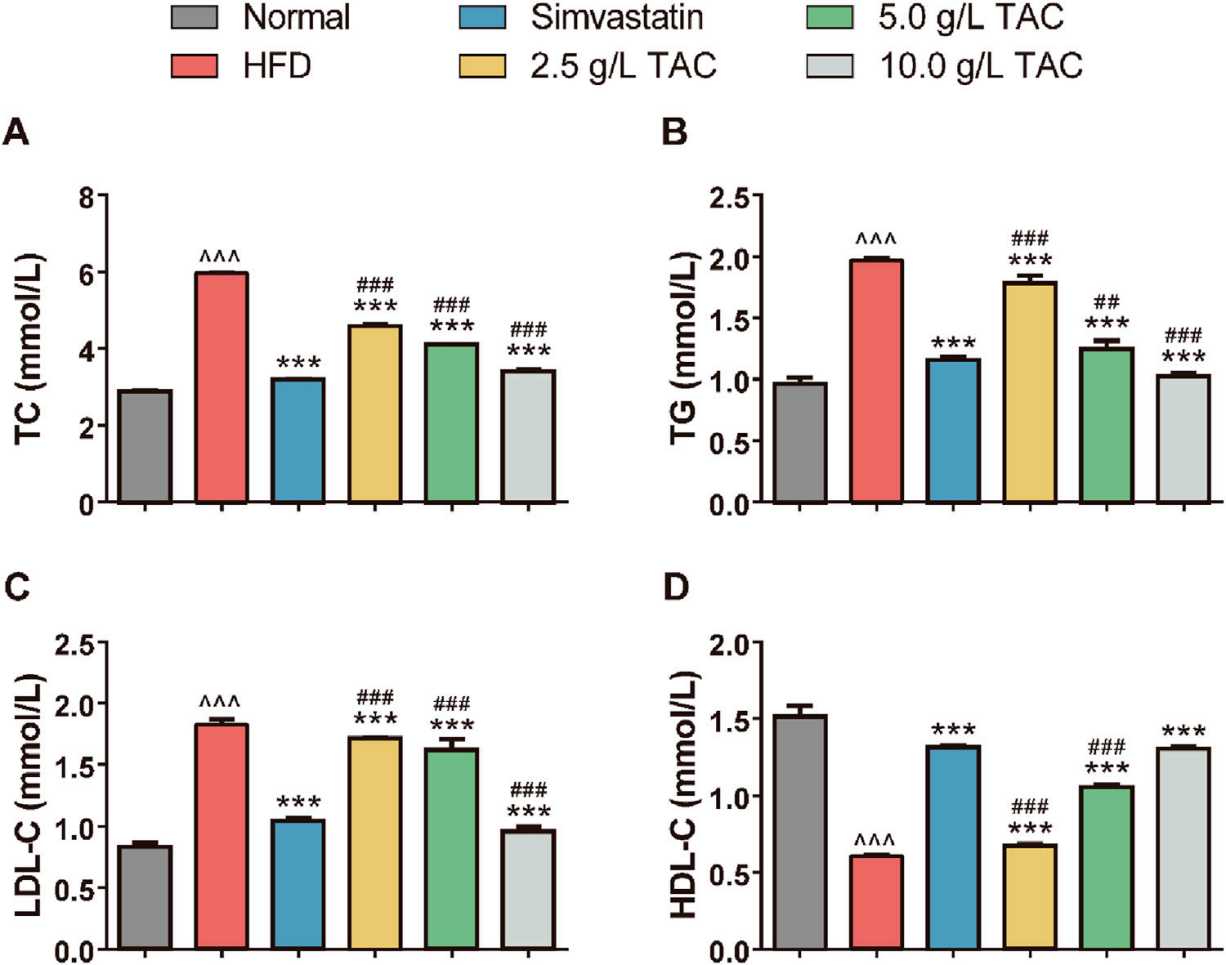
Effects of TAC on blood lipid indices in hyperlipidemic fatty liver mice. Effects of different doses of TAC on **(A)** TC, **(B)** TG, **(C)** LDL-C, and **(D)** HDL-C. Data are presented as mean ± SD, *n* = 9. ^^^P < 0.001 vs. Normal group; ***P < 0.001 vs. HFD group; ^##^P < 0.01, ^###^P < 0.001 vs. simvastatin group.

Further analysis of the effects of TAC on blood lipid levels in hyperlipidemic fatty liver mice showed that a high-fat diet significantly increased serum TC, TG, and LDL-C levels while decreasing HDL-C levels (Figure 2). Following TAC intervention, the treatment groups exhibited significant reductions in serum TC, TG, and LDL-C levels and a significant increase in HDL-C level compared with the model control group (all *P < 0.001), with a positive correlation with TAC dose, indicating the therapeutic effect of TAC on hyperlipidemia-combined fatty liver in mice.

### 3.3 Effects of TAC on atherosclerosis indices (AI1, AI2) and liver function in mice

**FIGURE 3:**
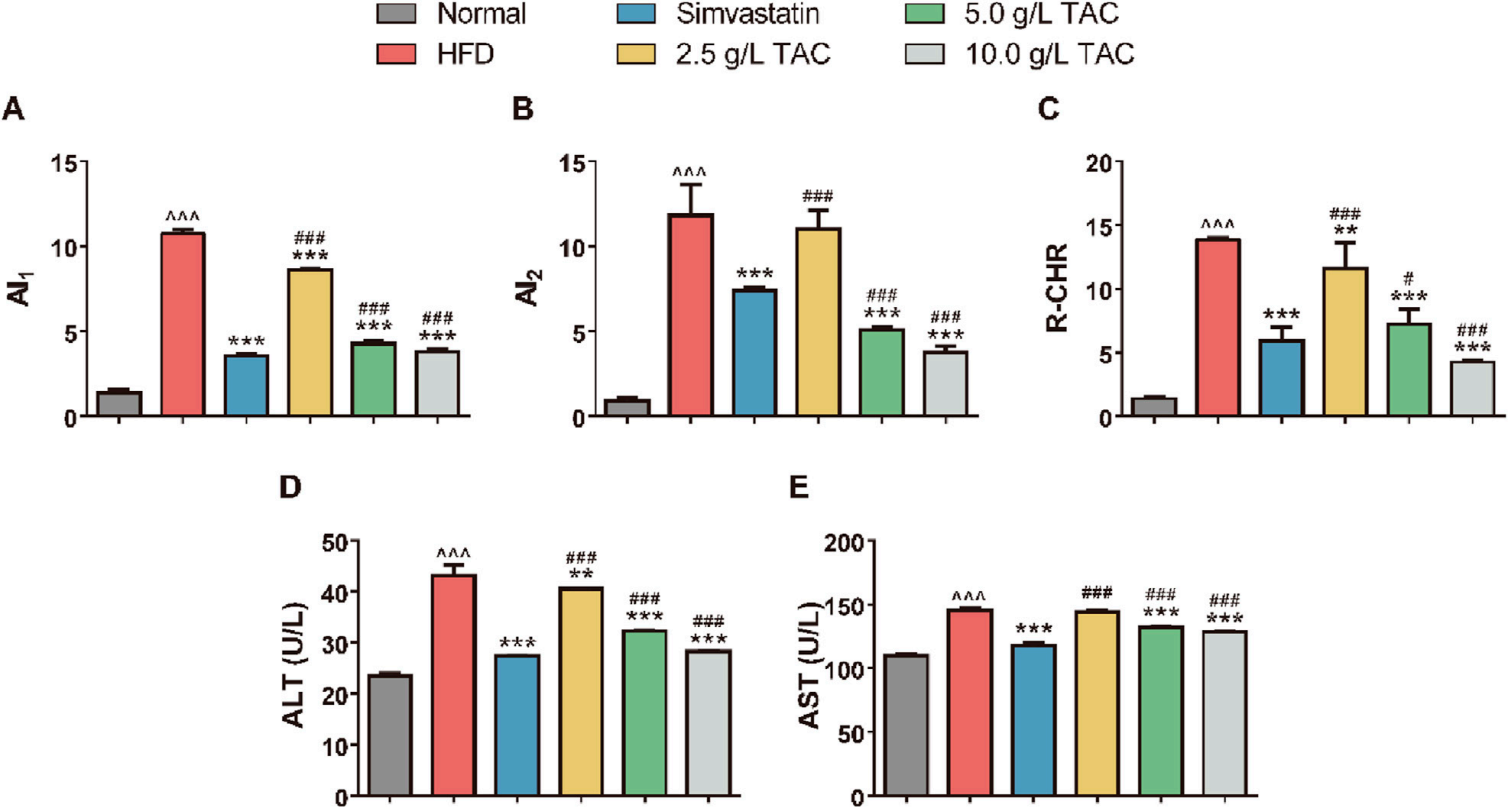
Regulatory effects of TAC on lipid metabolism and liver function in hyperlipidemic fatty liver mice. Effects of different doses of TAC on **(A)** AI1, **(B)** AI2, **(C)** R-CHR, **(D)** ALT, and **(E)** AST. Data are presented as mean ± SD, *n* = 9. ^^^P < 0.001 vs. Normal group; **P < 0.01, ***P < 0.001 vs. HFD group; ^##^P < 0.01, ^###^P < 0.001 vs. simvastatin group.

Compared with the normal group, the model control group showed significantly higher atherosclerosis indices (AI1, AI2) and coronary heart risk index (R-CHR) (Figure 3). In contrast, the simvastatin group and the medium- and high-dose TAC groups exhibited significant reductions in these indices (*P < 0.05), suggesting that TAC can reduce the risk of atherosclerosis. Notably, the AI2 and R-CHR levels in the high-dose TAC group were significantly lower than those in the simvastatin group (all *P < 0.001). Additionally, serum ALT and AST levels were significantly higher in the model group than in the normal group, indicating hepatic cell injury. In contrast, the medium- and high-dose TAC groups showed significant decreases in serum ALT and AST levels compared with the model group (all *P < 0.001), suggesting that TAC can improve liver function and exert hepatoprotective effects.

### 3.4 Effects of TAC on HL, LPL, and HMGCR activities and liver pathological changes in mice with hyperlipidemia and fatty liver

**FIGURE 4:**
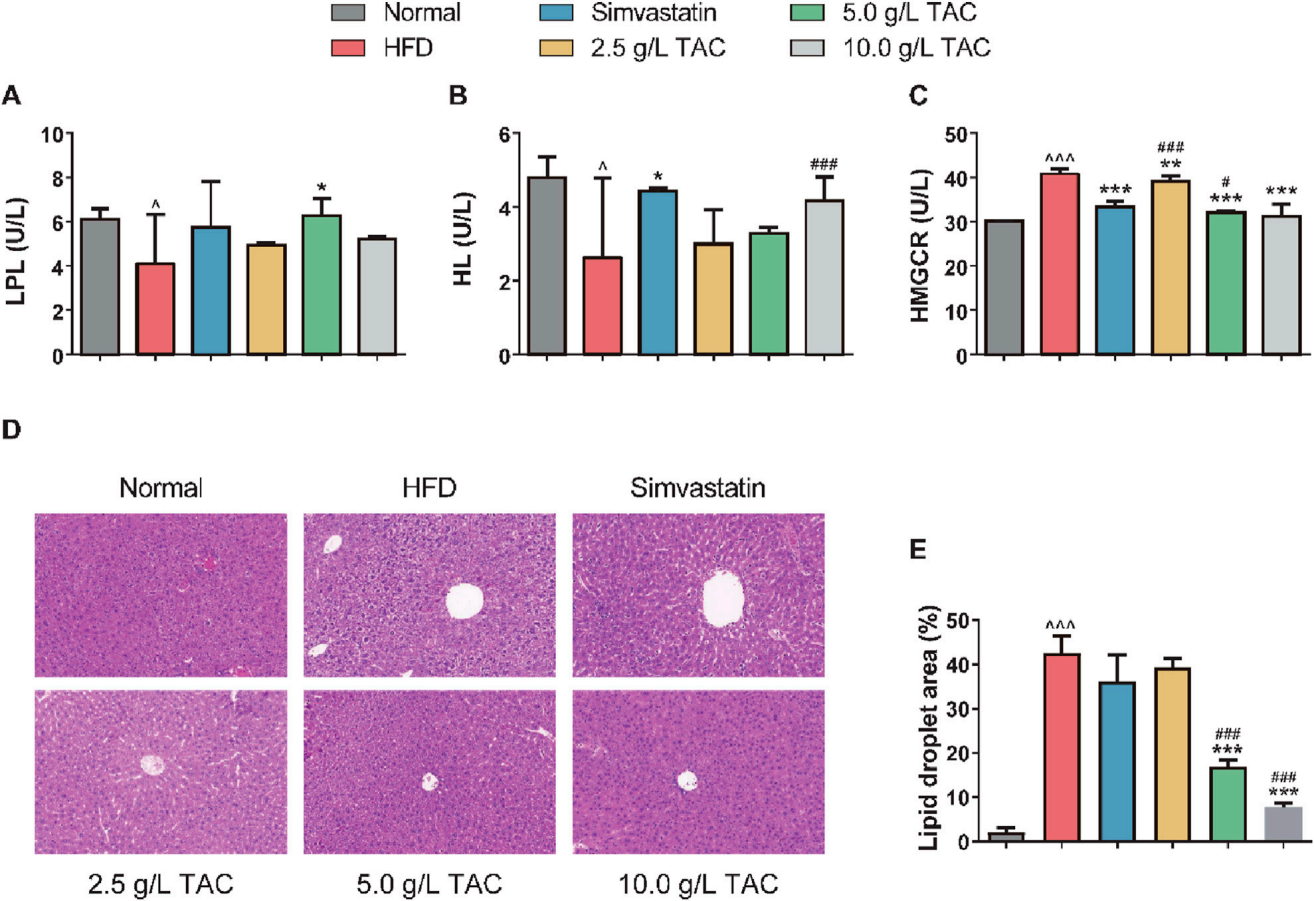
Effects of TAC on hepatic lipid metabolism factors and liver histopathology in hyperlipidemic fatty liver mice. Changes in **(A)** LPL, **(B)** HL, and **(C)** HMGCR levels, **(D)** hepatic histopathological changes and **(E)** Quantitative analysis of hepatic steatosis expressed as percentage of lipid droplet area by H&E staining (×200) in HFD mice treated with different doses of TAC. Data are presented as mean ± SD, *n* = 9. ^P < 0.05, ^^^P < 0.001 vs. Normal group; *P < 0.05, **P < 0.01, ***P < 0.001 vs. HFD group; ^#^P < 0.05, ^###^P < 0.001 vs. simvastatin group.

Further analysis of the effects of TAC on serum lipid metabolism enzyme activities showed that the model group had significantly reduced serum HL and LPL levels and significantly increased HMGCR levels, indicating the involvement of HL, LPL, and HMGCR in the development of hyperlipidemia and fatty liver (Figure 4). Compared with the model group, the 5.0 g/L TAC group showed a significant increase in serum LPL level (*P < 0.05). However, the low- and high-dose TAC groups showed no statistically significant differences in serum LPL levels compared with the model group (both *P* >0.05). Additionally, all TAC intervention groups had higher HL levels than the model group, but the differences were not statistically significant (all *P* >0.05). In contrast, all TAC intervention groups showed significant reductions in HMGCR levels compared with the model group (all *P < 0.01). H&E staining of liver tissues showed that the normal group had no significant pathological changes, with hepatocytes closely and uniformly arranged and intact cellular structures without obvious lipid droplets. In contrast, the model control group showed obvious cellular degeneration, hepatocyte swelling, disordered arrangement, and numerous fat droplets. Compared with the model control group, the high-, medium-, and low-dose TAC groups and the simvastatin group showed trends of reduced pathological changes, with some groups showing results similar to those of the simvastatin group, indicating therapeutic effects of TAC on the liver of mice with hyperlipidemia and fatty liver.

### 3.5 Effects of TAC on expression of lipid metabolism-related genes in mice

**FIGURE 5:**
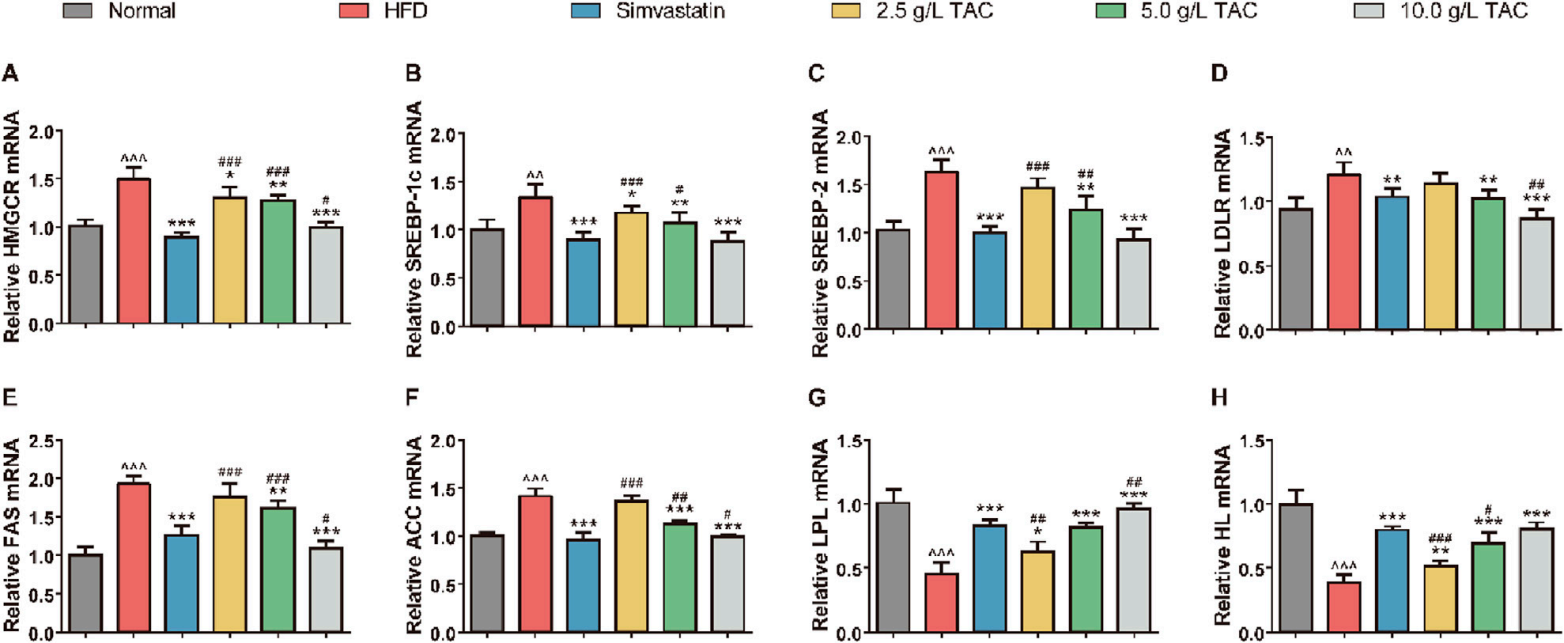
Effects of TAC on mRNA expression of hepatic lipid metabolism genes in hyperlipidemic fatty liver mice. qPCR quantification of mRNA levels of **(A)** HMGCR, **(B)** SREBP-1c, **(C)** SREBP-2, **(D)** LDLR, **(E)** FAS, **(F)** ACC, **(G)** LPL, and **(H)** HL. Data are presented as mean ± standard deviation (*n* = 9). Data are presented as mean ± SD, *n* = 9. ^^P < 0.01, ^^^P < 0.001 vs. Normal group; *P < 0.05, **P < 0.01, ***P < 0.001 vs. HFD group; ^#^P < 0.05, ^##^P < 0.01, ^###^P < 0.001 vs. simvastatin group.

To determine whether TAC-induced changes in serum lipid metabolism and improvement in liver fat accumulation were associated with alterations in lipid compensation in mice, qPCR was used to detect the expression levels of lipid metabolism-related genes in the liver of HFD mice (Figure 5). The results showed significant disorders in the expression profile of hepatic lipid metabolism genes in HFD mice. Compared with the normal group, the HFD group showed significantly upregulated expression of HMGCR, SREBP-1c, SREBP-2, and LDLR. Additionally, key genes for fatty acid synthesis (FAS and ACC) were simultaneously upregulated, while expression of rate-limiting enzymes for lipolysis (LPL and HL) was significantly suppressed, collectively constituting the molecular basis for hepatic lipid accumulation (Figures 5A–H). Following intervention with different doses of TAC, the above abnormalities were reversed in a dose-dependent manner. Notably, intervention with 10.0 g/L TAC restored the expression of all the above factors to normal levels (all *P < 0.001). Importantly, 10.0 g/L TAC significantly downregulated LDLR and FAS expression and upregulated LPL expression, with effects significantly stronger than those of the simvastatin group (*P < 0.05).

### 3.6 Molecular mechanism of TAC-regulated HMGCR

**FIGURE 6:**
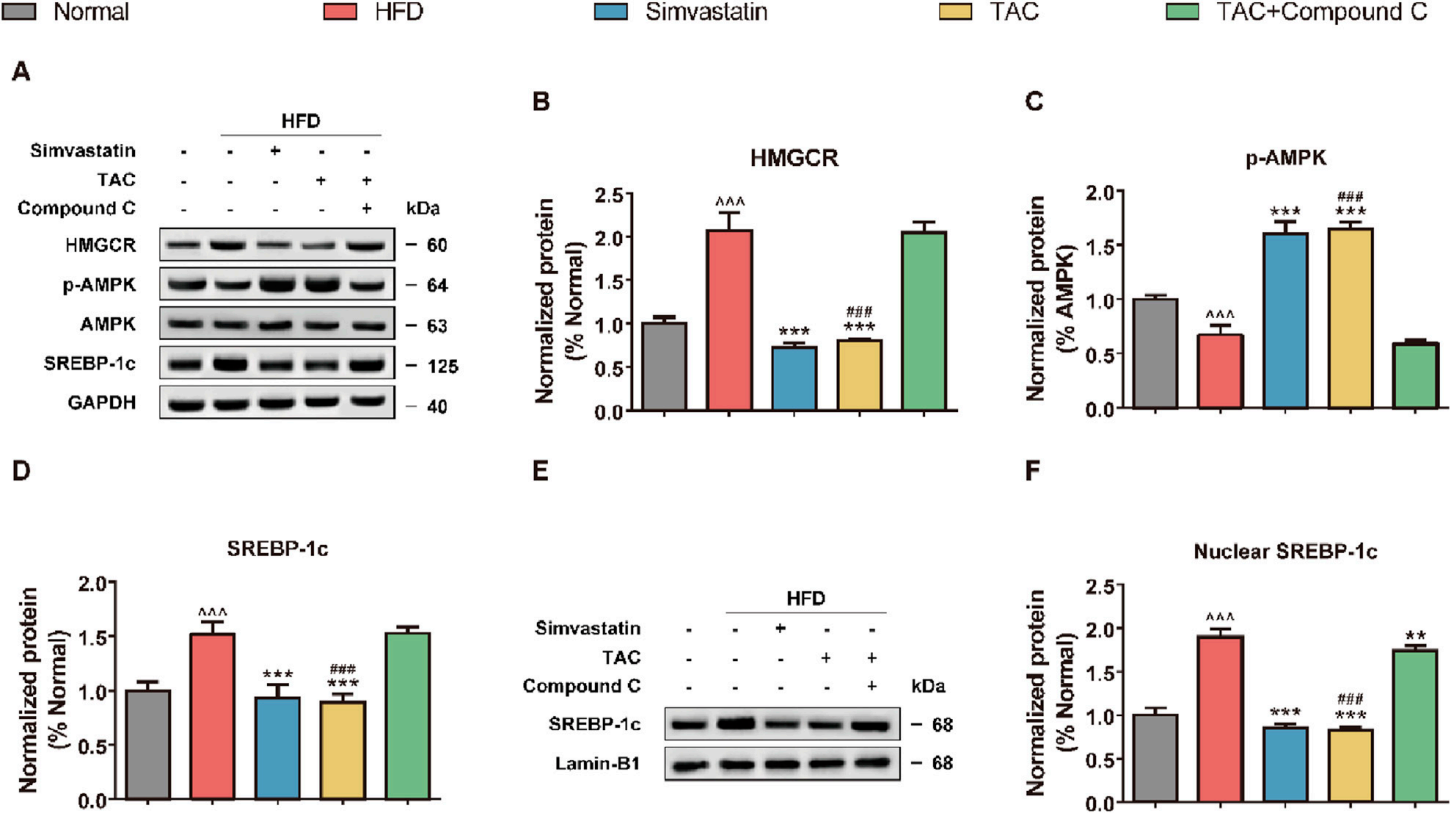
TAC regulates hepatic lipid synthesis-related protein expression via the AMPK/SREBP-1c pathway and validation of Compound C blocking effect in HFD mice. **(A)** Representative Western blot images of the AMPK/SREBP-1c pathway and **(E)** nuclear SREBP-1c, and protein expression levels of **(B)** HMGCR, **(C)** p-AMPK, **(D)** SREBP-1c, and **(F)** nuclear SREBP-1c. Data are presented as mean ± SD, *n* = 5. ^^^P < 0.001 vs. Normal group; **P < 0.01, ***P < 0.001 vs. HFD group; ^###^P < 0.001 vs. TAC+Compound C group.

Western blot analysis was further performed to assess the activation status of key proteins in the AMPK/SREBP-1c pathway in the liver tissue of HFD mice, to clarify whether TAC inhibits SREBP-1c nuclear translocation via AMPK (Figure 6). The results showed that a high-fat diet significantly suppressed hepatic AMPK phosphorylation and activated HMGCR (a key enzyme for cholesterol synthesis) and SREBP-1c (a transcription factor for fatty acid synthesis). Following TAC intervention, phosphorylated AMPK levels increased significantly (*P < 0.001), while HMGCR and SREBP-1c levels decreased significantly (both *P < 0.001). Interestingly, compared with the HFD group, the nuclear SREBP-1c protein abundance in the TAC group was significantly reduced (*P < 0.001), suggesting that TAC significantly inhibits SREBP-1c nuclear translocation. When an AMPK-specific inhibitor Compound C was used to block the pathway, the protective effects of TAC were completely abolished. The p-AMPK/AMPK ratio in the TAC+Compound C group was significantly reduced by 52% compared with the TAC group (*P < 0.001), and nuclear SREBP-1c accumulation and HMGCR expression both increased significantly (both *P < 0.001). These results indicate that TAC inhibits SREBP-1c nuclear translocation by activating AMPK phosphorylation.

### 3.7 Regulatory mechanism of TAC on LPL/HL activity

**FIGURE 7:**
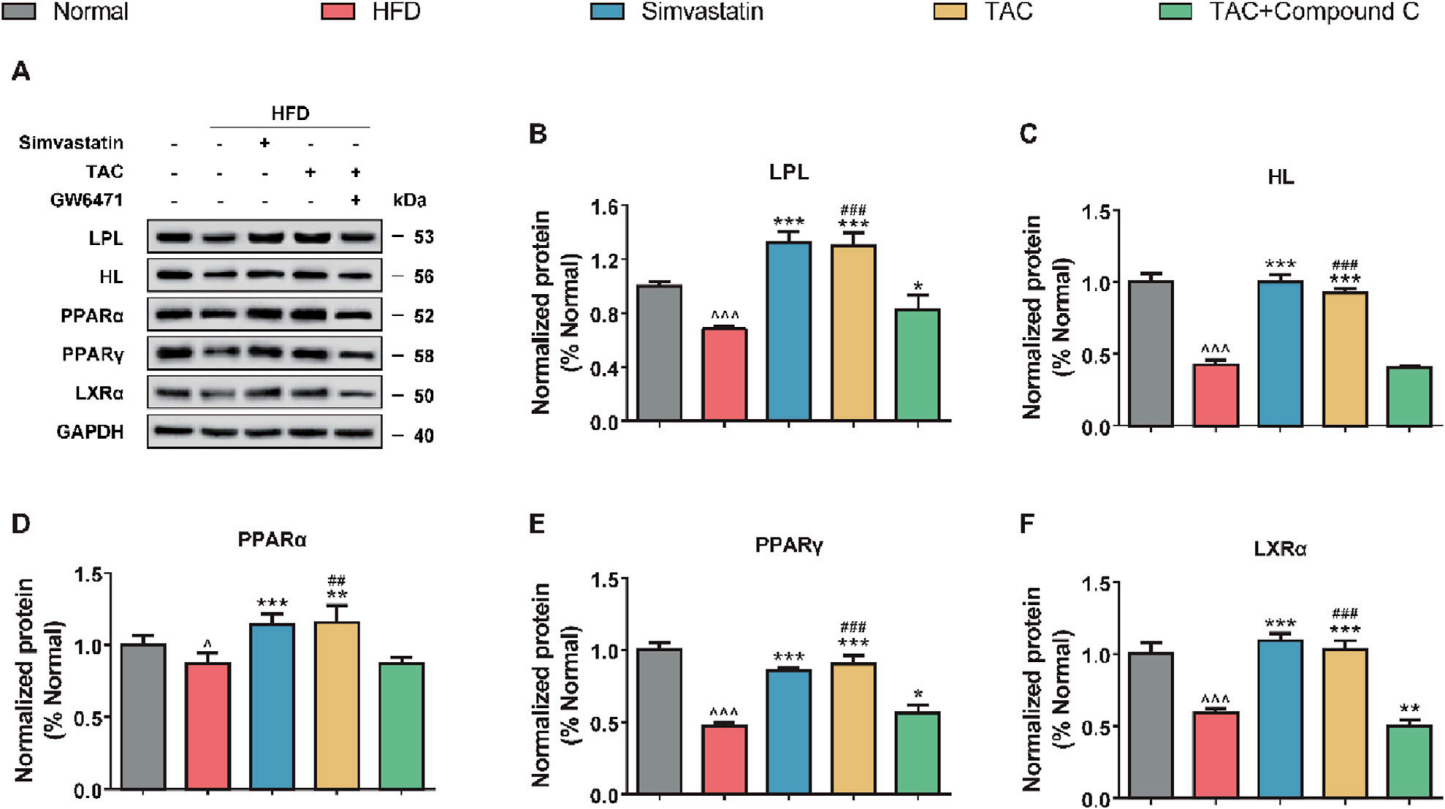
TAC regulates hepatic lipolysis-related protein expression via the PPARα/LXRα axis and validation of GW6471 blocking effect in HFD mice. **(A)** Representative Western blot images and protein expression levels of **(B)** LPL, **(C)** HL, **(D)** PPARα, **(E)** PPARγ, and **(F)** LXRα. Data are presented as mean ± SD, n = 5. ^P < 0.05, ^^^P < 0.001 vs. Normal group; *P < 0.05, **P < 0.01, ***P < 0.001 vs. HFD group; ^##^P < 0.01, ^###^P < 0.001 vs. TAC+GW6471 group.

Further exploration of the effects of TAC on LPL/HL and related transcription factors (PPARα, PPARγ) showed that a high-fat diet significantly suppressed the expression of key proteins in the hepatic lipolysis pathway, including LPL, HL, PPARα, PPARγ, and LXRα (Figure 7). Following TAC intervention, the expression of these factors was significantly restored (all *P < 0.001). When a PPARα-specific inhibitor GW6471 was used to block the pathway, the above effects of TAC were significantly reversed (all *P < 0.01), suggesting that TAC regulates LPL and HL expression via the PPARα/LXRα axis.

## 4 Discussion

The liver serves as the central hub for lipid homeostasis, and hepatic steatosis represents a paradigm of dysregulated hepatic lipid metabolism (Yip et al., 2023). Clinically, 20%–81% of fatty liver cases are complicated by hyperlipidemia, with epidemiological evidence indicating that high-fat diet consumption exacerbates hyperlipidemia risk. This metabolic derangement arises from aberrant lipid metabolism, leading to increased blood viscosity and decelerated blood flow, which underpin the pathogenesis of cardiovascular and cerebrovascular diseases (Taha et al., 2021). The rising global incidence of these disorders underscores the critical need for novel therapeutic strategies to improve patient outcomes (Alloubani et al., 2021). *Coptis chinensis* Franch (TAC), a traditional Chinese herbal medicine, exhibits heat-clearing, dampness-resolving, and detoxifying properties, alongside well-documented safe lipid-lowering effects (Li et al., 2023). This study established a murine model of hyperlipidemia with hepatic steatosis to characterize the regulatory effects of total alkaloids from TAC on lipid metabolism, hepatic function, and histopathology. Results demonstrated that TAC significantly ameliorated high-fat diet-induced increases in body weight, liver wet weight, and hepatic coefficient, normalized fasting glucose, and regulated serum lipid profiles (TC, TG, LDL-C, HDL-C) and hepatic enzymes (ALT, AST). Mechanistically, TAC alleviated hepatic lipid accumulation by modulating the activity of key lipometabolic enzymes (HL, LPL, HMGCR), thereby exerting therapeutic effects against hyperlipidemia-combined hepatic steatosis.

The model group exhibited profound dyslipidemia, with significantly elevated TC, TG, and LDL-C levels and reduced HDL-C, consistent with high-fat diet-induced metabolic derangement. TAC intervention, particularly at high doses, normalized lipid parameters comparably to simvastatin, aligning with prior studies showing that berberine, the major alkaloid component of *C. chinensis*, reduces hepatic lipid anabolism and promotes catabolism by activating the AMPK pathway to inhibit fatty acid synthase (FAS) expression (Kong et al., 2009). TAC likely enhances triglyceride clearance via upregulation of LPL and HL activity, complementing its lipid-lowering effects. The model group showed marked hepatic injury, as evidenced by elevated ALT and AST levels, which were significantly mitigated by TAC, suggesting hepatoprotective effects possibly mediated by *C. chinensis*-derived antioxidant and anti-inflammatory properties. TAC also inhibited HMGCR activity, reducing cholesterol biosynthesis to protect hepatocytes, analogous to statin-class medications (Balraj et al., 2023). Histopathological analysis via H&E staining revealed severe steatosis and inflammatory infiltration in the model group, which were substantially ameliorated by TAC, consistent with improved biochemical indices. These findings parallel reports that berberine improves NAFLD pathology by regulating lipid metabolism and suppressing oxidative stress (Yan et al., 2015).

Building on TAC’s efficacy in improving serum lipids and hepatic function, we interrogated its impact on hepatic lipid metabolism gene expression in HFD mice. HMGCR, the rate-limiting enzyme of cholesterol biosynthesis, directly dictates cholesterol production (Pierzchlińska et al., 2021). Sterol regulatory element-binding proteins (SREBP-1c and SREBP-2) serve as master transcriptional regulators of fatty acid and cholesterol synthesis, respectively (Shi et al., 2025; Walker et al., 2011). SREBP-1c primarily orchestrates fatty acid anabolism, while SREBP-2 governs cholesterol synthesis gene expression (Walker et al., 2011). Low-density lipoprotein receptor (LDLR), transcriptionally regulated by SREBP-2, mediates circulating LDL-C clearance in the liver (Chandran et al., 2025). Key enzymes of fatty acid synthesis, fatty acid synthase (FAS) and acetyl-CoA carboxylase (ACC), are also under SREBP-1c control (Xiong et al., 2025). Preclinical studies have shown that environmental toxicants (e.g., bisphenol A) activate HMGCR-SREBP-2 signaling to augment cholesterol synthesis (Li et al., 2019), while natural compounds like acetyl-L-carnitine and flavonoids ameliorate atherosclerotic phenotypes by inhibiting SREBP-2-mediated cholesterol biosynthesis (Xing et al., 2024; Wu et al., 2021). Collectively, these findings highlight the intricate regulatory network of HMGCR, SREBP-1c, SREBP-2, LDLR, FAS, and ACC in hepatic metabolism, identifying potential therapeutic nodes for metabolic disorders. Our data showed that TAC significantly downregulated these factors in HFD mice, indicating restoration of dysregulated hepatic lipid metabolism in hyperlipidemia and NAFLD.

Elucidating the AMPK/SREBP-1c pathway in HFD mice is pivotal for understanding TAC’s mechanistic action (Zhu et al., 2019). As a master regulator of cellular energy homeostasis, AMPK curbs lipid anabolism by inhibiting SREBP-1c nuclear translocation, thereby reducing hepatic lipid accumulation (Zhu et al., 2019). AMPK activation suppresses SREBP-1c maturation and nuclear shuttling, decreasing expression of lipid synthesis genes. For instance, berberine inhibits SREBP-1c proteolytic processing and nuclear translocation via AMPK, reducing lipid synthetic gene expression and intracellular lipid droplet formation (Jang et al., 2017). Similarly, deoxypodophyllotoxin reduces lipid synthesis by activating AMPK to suppress SREBP-1c (Kim et al., 2018). AMPK signaling also regulates lipid metabolism through alternative pathways; isosilybin improves hepatic lipid handling by modulating the AMPK/SREBP-1c/PPARα axis to balance lipid synthesis and β-oxidation (Liu et al., 2022). Cumulative evidence shows that AMPK activation not only restrains lipid anabolism but also promotes fatty acid oxidation (Kim et al., 2019). *Scutellaria baicalensis* ameliorates NAFLD via AMPK-mediated SREBP signaling (Chen et al., 2018), aligning with our findings that TAC significantly enhanced hepatic AMPK phosphorylation in HFD mice while suppressing HMGCR and SREBP-1c expression, including nuclear SREBP-1c. These effects were abrogated by an AMPK-specific inhibitor, confirming that TAC regulates lipid metabolism through AMPK phosphorylation-dependent inhibition of SREBP-1c nuclear translocation and promotion of fatty acid oxidation.

Hepatic lipase (HL) and lipoprotein lipase (LPL), members of the triglyceride lipase family, play indispensable roles in lipoprotein metabolism (Shang and Rodrigues, 2021). LPL, predominantly expressed in adipose tissue, myocardium, and skeletal muscle, hydrolyzes triglycerides in chylomicrons (CM) and very low-density lipoproteins (VLDL) to release free fatty acids for peripheral tissue utilization (Sylvers-Davie and Davies, 2021). Under physiological conditions, LPL efficiently decomposes plasma triglycerides, but its activity is often compromised in hyperlipidemia (Wu et al., 2023). In cardiac tissue, LPL supplies fatty acids for ATP production via lipoprotein triglyceride hydrolysis, underscoring its role in myocardial energy metabolism (Shang and Rodrigues, 2021). Like LPL, HL is involved in systemic lipid homeostasis, primarily expressed in the liver to facilitate triglyceride hydrolysis and lipoprotein remodeling (Koerner et al., 2019). While HL and LPL share functional similarities, they exhibit tissue-specific specializations: HL mediates hepatic triglyceride metabolism, whereas LPL supports energy supply in cardiac and skeletal muscle (Shang and Rodrigues, 2021). Regulation of LPL and HL activity is critical for maintaining lipid metabolic balance, with their dysregulation contributing to hyperlipidemia pathogenesis.

**FIGURE 8:**
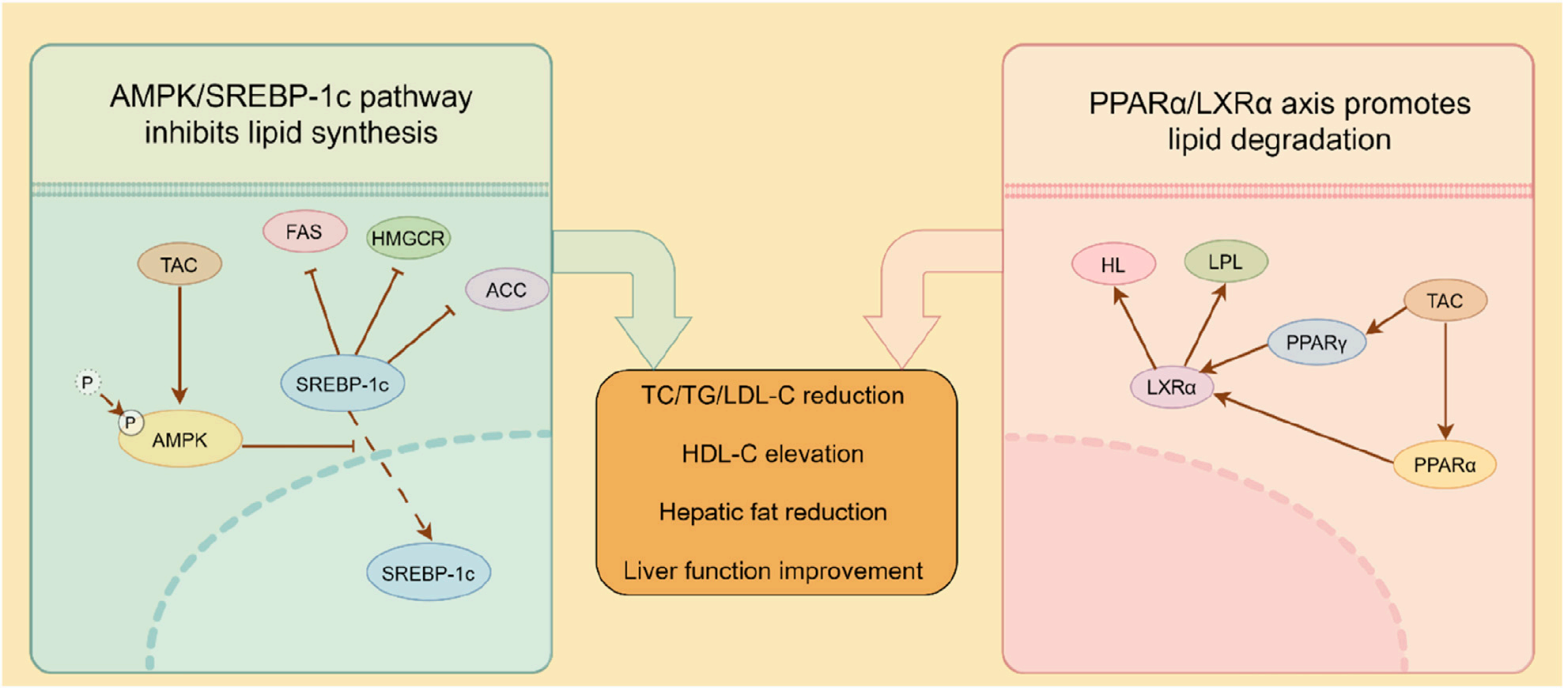
Proposed dual-pathway regulatory mechanism of TAC demonstrated in mice.

This study revealed that reduced serum HL and LPL activity in model mice impaired triglyceride catabolism, leading to lipid accumulation and exacerbation of hyperlipidemia. TAC significantly upregulated HL and LPL activity to promote triglyceride hydrolysis, consistent with prior reports (An et al., 2022). Preclinical evidence shows that enhancing LPL activity improves triglyceride clearance and lipid profile modulation, underscoring its therapeutic potential in hyperlipidemia (Goldberg and Merkel, 2001). Reduced HL activity compromises LDL clearance, contributing to hypercholesterolemia and hypertriglyceridemia, hallmarks of metabolic syndrome. Decreased HL levels in hyperlipidemic fatty liver mice further implicate HL in disease progression. Strategies to enhance HL activity or expression promote lipoprotein metabolism and reduce circulating lipids, offering therapeutic promise (Goldberg and Merkel, 2001). Additionally, TAC upregulated hepatic HL and LPL expression. Investigation of key lipolytic pathway proteins (PPARα, PPARγ, LXRα) in HFD livers showed that PPARα, a nuclear receptor, regulates lipid metabolism and energy balance by modulating gene expression (Fougerat et al., 2024). PPARα activation promotes fatty acid oxidation and ketogenesis to maintain energy homeostasis during fasting (Fougerat et al., 2024) and regulates autophagy-lysosome function, impacting cellular bioenergetics (Sinha et al., 2020). PPARγ overexpression induces hepatic steatosis (Kim et al., 2017), while its activation is linked to lipid accumulation and inflammatory signaling (Jie et al., 2021). As another nuclear receptor, LXRα regulates cholesterol homeostasis and lipid synthesis, with activation influencing membrane biophysics via polyunsaturated fatty acid metabolism (Jalil et al., 2019). LXRα-PPARγ crosstalk modulates lipid metabolism and inflammatory responses (Palermo et al., 2016). Overall, a proposed mechanism mediated by TAC was established as shown in Figure 8, in which TAC intervention upregulated LPL, HL, PPARα, PPARγ, and LXRα expression, an effect reversed by a PPARα inhibitor, establishing that TAC regulates LPL and HL via the PPARα/LXRα axis.

While the use of specific inhibitors (Compound C for AMPK and GW6471 for PPARα) provides strong functional evidence supporting our proposed mechanism, we acknowledge the inherent limitation that pharmacological agents can have off-target effects. Therefore, the complete abolition of TAC’s effects upon co-treatment with these inhibitors could theoretically be influenced, in part, by actions on unintended targets. However, several aspects of our data bolster confidence in our conclusions. First, the effects are pathway-specific: Compound C selectively reversed TAC’s impact on the AMPK/SREBP-1c axis, while GW6471 specifically blocked its effects on the PPARα/LXRα axis. This mutual exclusivity suggests that the observed outcomes are not due to a common, nonspecific toxic effect. Second, the convergence of evidence is compelling: The fact that two chemically distinct inhibitors, targeting two entirely different pathways, each completely abrogated TAC’s efficacy in a mutually exclusive manner provides a strong, self-consistent argument for the involvement of both pathways. Future studies employing genetic approaches, such as siRNA or CRISPR/Cas9-mediated knockout of AMPK or PPARα in hepatocytes, would be invaluable to conclusively confirm these findings without the potential confound of pharmacological off-target effects. Furthermore, the inhibition of AMPK by Compound C not only suppressed its own downstream targets but also markedly reduced the protein levels of PPARα and LXRα. This observation suggests that the activation of the PPARα/LXRα axis by TAC may be partially dependent on prior AMPK activation. Therefore, the dual modulation we observed may not be entirely independent; instead, AMPK may act as an upstream regulator that primes and enhances the PPARα signaling pathway, creating a powerful, self-reinforcing metabolic cascade that potently drives lipid catabolism and suppresses synthesis. This hypothesized crosstalk provides a sophisticated mechanistic model for TAC’s efficacy and represents a fascinating area for future investigation.

The present study employed a broad dose range of TAC (2.5–10.0 g/L) to capture its therapeutic window. A careful examination of our results reveals a compelling dose-dependent relationship for the majority of the measured parameters. Key metrics such as body weight reduction, improvement in serum lipid profiles (TC, TG, LDL-C), and normalization of lipid metabolism gene expression (e.g., HMGCR, SREBP-1c, LPL) exhibited a clear trend of enhanced efficacy with increasing TAC concentration. Notably, for several critical outcomes, the medium dose (5.0 g/L) emerged with the most favorable profile. For instance, the elevation of HDL-C and the reduction of atherosclerosis indices (AI1, AI2) and coronary risk index (R-CHR) were most pronounced at the 5.0 g/L dose, with its effects often being comparable or even superior to the highest dose (10.0 g/L). This phenomenon suggests that while the high dose is effective, the medium dose (5.0 g/L) may represent the optimal effective dose within our experimental paradigm, achieving maximal therapeutic benefit without incurring potential diminished returns that can sometimes occur at the upper end of a dose-response curve. This optimal dose provides a crucial reference for future mechanistic studies and potential translational development.

A critical question is whether the observed therapeutic effects are unique to the total alkaloids (TAC) or can be primarily attributed to berberine (BBR), its most abundant (∼70%–80%) and well-studied component. The superiority of a multi-component extract may lie in multi-target synergy, where different alkaloids concurrently modulate complementary nodes in the metabolic network (e.g., AMPK by BBR and coptisine, PPARα by BBR and palmatine). This polypharmacological approach can enhance overall efficacy and reduce the likelihood of compensatory mechanisms that can limit the effects of single-target agents. Therefore, TAC represents a holistic therapeutic strategy that leverages the concerted action of its constituents, potentially offering a more robust and sustainable outcome for managing complex metabolic syndromes than berberine monotherapy.

This study harbors several limitations warranting further investigation. Firstly, while the dual-pathway regulatory mechanism of TAC was demonstrated in mice, its clinical translation requires validation in higher-order animal models. Species-specific differences in lipid metabolic networking between rodents and humans necessitate clinical verification of TAC’s effects on human HMGCR activity and the PPARα/LXRα axis (Yip et al., 2023; Yan et al., 2015). Secondly, while this study elucidates the integrated effect of the total alkaloids, the potential synergistic interactions between its constituent alkaloids (e.g., berberine, coptisine, palmatine) and a direct mechanistic comparison with purified berberine remain to be fully explored (Yang et al., 2024; Kong et al., 2009). Furthermore, while our data strongly imply enhanced fatty acid oxidation through the AMPK-ACC axis, direct measurement of downstream targets such as CPT1 activity and expression will be an important focus of our subsequent research to fully elucidate the scope of TAC’s impact on mitochondrial lipid metabolism. Additionally, the impact of TAC on the gut microbiota-lipid metabolism axis was not explored, despite prior evidence that Coptis alkaloids inhibit trimethylamine N-oxide (TMAO) production via microbiota regulation (Li et al., 2021), a potential pathway for TAC’s anti-atherosclerotic effects. Finally, the study’s short-term intervention lacked long-term toxicity assessment and dynamic monitoring of liver fibrosis progression, limiting comprehensive evaluation of its clinical applicability.

In conclusion, TAC’s dual-pathway regulation (promoting lipolysis and inhibiting synthesis) provides a theoretical basis for treating hyperlipidemia with fatty liver. Future studies should integrate metabolomics and metagenomics to dissect TAC’s multi-target regulatory network, and conduct dose-dependent long-term toxicity trials to facilitate clinical translation.

## Data Availability

The original contributions presented in the study are included in the article/supplementary material, further inquiries can be directed to the corresponding author.
